# Does the Surgical Safety Checklist need a co-pilot? Comparing adherence in gynecological surgery through electronic medical records and OR Black Box video observations

**DOI:** 10.1007/s00464-025-11966-0

**Published:** 2025-07-17

**Authors:** Kjestine Emilie Møller, Jette Led Sørensen, Susanne Rosthøj, Patricia Trbovich, Teodor Grantcharov, Jeanett Strandbygaard

**Affiliations:** 1 Department of Gynecology, Fertility and Obstetrics, Copenhagen University Hospital, Rigshospitalet, Blegdamsvej 9, 2100 Copenhagen, Denmark; 2 Juliane Marie Centre, Children’s Hospital Copenhagen, Copenhagen University Hospital, Rigshospitalet, Copenhagen, Denmark; 3 Department of Clinical Medicine, Faculty of Health and Medical Sciences, University of Copenhagen, Copenhagen, Denmark; 4 Mary Elizabeth’s Hospital – Rigshospitalet for Children, Teens and Expecting Families, Copenhagen, Denmark; 5 Danish Cancer Institute, Statistics and Data Analysis, Copenhagen, Denmark; 6 Institute of Health Policy, Management and Evaluation, University of Toronto, Toronto, ON Canada; 7 North York General Hospital, Toronto, ON Canada; 8 Department of Surgery, Clinical Excellence Research Centre, Stanford University, Stanford, CA USA

**Keywords:** Surgical Safety Checklist, Operating Room Black Box, Safety, Adherence, Observational study

## Abstract

**Background:**

Despite clear evidence that the Surgical Safety Checklist improves patient safety, the way its use is reported in the literature varies significantly. Consequently, we must understand the alignment between reported use of the checklist and its actual application to identify discrepancies that could affect safety reporting accuracy, and ultimately, patient safety outcomes. The study aims to examine Surgical Safety Checklist adherence in a gynecological operating room based on video data and to compare the resulting findings with reported use in patient electronic medical records.

**Method:**

An observational study was conducted on elective gynecological surgeries in a single operating room equipped with an OR Black Box from August to October 2021 to assess checklist compliance, quality, and engagement. The checklist’s reported use in patient electronic medical records was reviewed.

**Results:**

Forty-five surgeries were assessed. The video observed compliance score for Sign-in and Time-out was 100%, but 80% for Sign-out. Engagement scores, i.e., percentage of people paused, varied during the three checklist phases, with an overall mean score of 76% (range 45–94%). Quality scores, i.e., percentage of checklist items completed, differed between video observed (47% (95% CI 43–50)) and electronic medical records reported (89% (95% CI 84–94)) use.

**Conclusions:**

OR Black Box video provides a unique opportunity to assess the actual use of the Surgical Safety Checklist, revealing valuable insights into *how* it was used. Data showed that the checklist was not used as intended. A discrepancy was found between the reported completion in the electronic medical records and its actual use as observed in the video, with the former showing a much higher completion rate. This large discrepancy highlights the need for further initiatives to improve checklist use and reporting.

**Supplementary Information:**

The online version contains supplementary material available at 10.1007/s00464-025-11966-0.

The World Health Organization (WHO) introduced the Surgical Safety Checklist (SSC) in 2008 as part of its Safe Surgery campaign [1] as a mandatory safety tool to support clinical practice and minimize the number of errors and adverse events during surgeries [2]. SSC has since been widely adopted worldwide [3], and is used by almost all surgical specialties and healthcare professional (HCP) groups (e.g., surgeons, anesthesiologists, operating room (OR) nurses, and nurse anesthetists) involved in surgical procedures. SSC ensures that certain procedural checks and communication prompts [4], such as critical and important information about the surgical team, procedure, and patient, are completed and verbalized to all HCP [1].

SSC is designed to be used in three different phases of the surgical procedure: Sign-in, sometimes referred to as Briefing, (before induction of anesthesia), Time-out (before skin incision), and Sign-out, sometimes referred to as Debriefing, (before patient leaves the OR).

Numerous studies have shown that using SSC clearly decreases mortality and morbidity rates [5–9]. Moreover, a recent study showed that solid execution of the checklist is associated with better patient outcomes [10]. However, other studies have not found the same positive impact [11], with implementation of SSC continuing to remain a challenge [12–14]. A wide array of factors across various levels of healthcare systems impact the SSC’s implementation and execution [13, 15].

Despite mandatory use of SSC in most ORs worldwide, observational studies have shown that the three SSC phases are only partially completed or not completed at all [16–21], and that HCPs are often not fully engaged or actively participating [17, 18]. This variation in use of the checklist, including when it is not used as intended, may affect patient safety [22, 23]. Much SSC research has focused on whether its introduction and use impact patient outcomes, e.g., mortality and morbidity [6].

Before investigating this association and ensuring the intended effectiveness of SSC, it is important to understand execution of the checklist and if the reported data provides a representative picture of checklist use. How the literature reports its use is not done uniformly [24]. Various approaches, such as reviewing medical records, conducting surveys, analyzing video footage, or observing procedures firsthand, have been used to evaluate checklist adherence. However, documentation types like questionnaires, and self-reported inputs may introduce bias, leading to missing details or an inflated sense of compliance [25]. Previous studies have highlighted significant discrepancies between real-time observations and reported accounts [16, 26, 27]. Currently, knowledge about execution of SSC at our institution is based on data reported in patient electronic medical records (EMRs).

Video-based observations are considered a powerful tool in evaluating clinical practices by providing an exact picture of what was said and done. Some of the advantages are that videos can be replayed as needed in full or in segments, and more than one observer can view them. In addition, videos can provide information about factors (e.g., communication, non-verbal language, and engagement among HCPs) that other methods do not necessarily capture [28]. The OR Black Box system, a multichannel technology that captures data from different sources, including video and audio data from the OR [29], is considered suitable for capturing objective data about the performance of the SSC inside the OR [17, 18], as it allows for the assessment of what was actually done. The OR Black Box has been in use at our institution since 2020 [30] and is currently implemented in several ORs.

This study investigates SSC adherence in a Danish gynecological-anesthesiologic OR, comparing observed performance via OR Black Box video with reported use in patient EMRs. The study evaluates how each phase and individual SSC item is completed, whether they are done at the correct time, and what level of staff engagement (correct pauses) occurs during the three SSC phases. The goal is to identify performance gaps so future interventions can be designed to improve SSC adherence.

## Methods and materials

### Study design and setting

We conducted this observational study of SSC adherence during elective gynecological surgeries at the Departments of Gynecology and Anaesthesiology, Copenhagen University Hospital – Rigshospitalet, Copenhagen, Denmark, from August to October 2021. The OR Black Box system, which is a multichannel technological platform that collects and compiles room view video and audio from the OR, patient vital signs, and data from various surgical equipment during surgery [29], was used to record the surgeries. A retrospective review of completed SSC items reported in patient EMRs was also conducted for each surgery.

Strengthening the Reporting of Observational Studies in Epidemiology guideline [31] was used to report this study.

### Subjects

The OR Black Box system was used to consecutively record forty-seven elective gynecological surgeries, with the room view video consequently used to assess what was said and done during the three SSC phases. All HCPs and patients present in the OR received oral and written information before recording began, and both patients and HCPs provided written consent prior to the data capture. If consent was not obtained, the OR Black Box was turned off.

### Data collection

#### Surgical Safety Checklist

The SSC [1] has been modified to fit local practices and requirements. Table S1 presents the original WHO SSC and the one modified for our surgical ward. At our department, the SSC is only visible on the computer screen in EMRs, and the circulating OR nurse is responsible for ticking off all checklist items.

Overall, the modified SSC comprises 28 items: 15 in the Sign-in phase; 8 in Time-out; and 5 in Sign-out (Fig. S1). Individual SSC items within each phase were categorized as procedural checks and communication prompts, in accordance with Singer et al. [4].

### Video assessment

Three members of the research group (KEM, JLS, and JS) assessed all video data at Surgical Safety Technologies in Toronto, Canada.

For each video, we registered the type of surgery, primary surgeon, and students and relatives present. We also noted timestamps for patients arriving in and leaving the OR, Sign-in start and end, induction and end of anesthesia, Time-out start and end, start of incision, closure, and Sign-out start and end, plus re-admission within 30 days post-surgery.

**Fig. 1:**
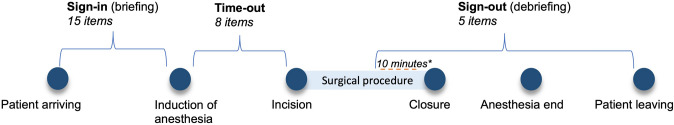
Timepoints during the surgical procedure and correct timing for the three Surgical Safety Checklist phases in the operating room. *Sign-out was considered executed if done between 10 min prior to closure and until the patient left the OR

All three phases (Sign-in, Time-out, and Sign-out) of the SSC were assessed for each surgery. Sign-in was considered performed if done in the period from the patient’s arrival in the OR until induction of anesthesia. All items were considered completed within this timeframe even when not necessarily completed consecutively in a structured way. Time-out was considered performed if done between induction of anesthesia, and start of incision. Last, Sign-out was considered performed if done 10 min prior to the start of closure or before patient leaving the OR (Fig. 1).

An item was considered completed if addressed at the correct time and verbalized by at least one HCP in the OR. If the item was not verbalized, it was considered as not completed.

The Sign-in items ‘Risk of airway problems’ and ‘Risk of aspiration’ were considered completed if relevant aspects were addressed, such as loose teeth, nothing by mouth, and heartburn, even though an item’s exact wording was not used.

The number of people present and focused was also registered for each surgery in each SSC phase. An HCP was considered focused if they paid attention and were not engaged with other tasks, e.g., opening instruments, putting gloves or surgical clothes on, using a phone, or engaged in conversation. We also registered who initiated and completed the various checklist phases based on the HCP’s role (anesthesiologist, gynecologist, nurse anesthetist, or OR nurse).

### Outcomes

Previous research using the OR Black Box [17, 18, 32] assessed SSC adherence based on the following metrics:

*Compliance:* indicates whether the three phases had been completed or not at the correct time.

*Quality:* measured as the percentage of individual checklist items completed within each of the three phases.

*Engagement:* measured as the percentage of focused (paused) participants out of total participants present in the OR during completion of the checklist.

These metrics cover *how* the checklist was performed, not just whether it was completed as a yes/no answer. Each SSC item was for both EMR and OR Black Box data, reported as a binary outcome, i.e., completed or not completed.

All scores for *compliance*, *quality*, and *engagement* are given on a scale from 0 to 100, with 100 representing the best and highest score.

It was possible to measure all three metrics for the data the OR Black Box captured. Assessing *engagement* was not possible for the EMR reported data, as no data were reported on who was participating and focused during the performance of the checklist, nor was it possible to determine whether SSC was adhered to at the correct time during surgical procedures, since these are not documented in the EMR system.

### Statistical analysis

Quantitative data were summarized using mean and median with interquartile range. Categorical variables were reported as frequencies and percentages. McNemar’s test was used to assess differences in paired binary data from the SSC. The proportion of identical scores between the data from EMR and OR Black Box data was determined with 95% Clopper-Pearson exact confidence intervals (CIs). The Wilcoxon signed-rank test was used to compare paired quality scores.

The general linear model was applied to explore differences in the mean quality scores and performed items, as well as differences in quality score and length of surgery. Multiple linear regression analyses, adjusted for the number of people present, were performed to test whether quality scores were associated with OR engagement scores.

All statistical analyses were performed using R version 4.2.0 [33], and *p*-values < 0.05 were considered statistically significant.

### Ethics and approvals

The Danish Data Protection Agency approved this study (VD-2019–275)/(P-2023–14538) but approval was not required from the Danish National Committee on Health Research Ethics. HCPs and patients gave oral and written consent to participate in the study. Departments of Gynecology and Anesthesiology management, Copenhagen University Hospital – Rigshospitalet approved the study.

## Results

The OR Black Box captured 47 gynecological surgical procedures during the consecutive two-month period that this study covered. Two surgeries were excluded, one due to technical issues regarding audio failure, and one due to patient withdrawal of consent, leaving 45 surgical procedures for assessment. Of these, 44 were elective surgeries, and one was an acute procedure – a return to the OR due to postoperative bleeding. Table 1 presents the descriptive details based on the OR Black Box data.

**Table 1:** Case characteristics based on OR Black Box video data

Other items of interest	Data
No. of cases	45
Type of surgery, *n* (%)	
Benign Oncology Endometriosis	11 (24) 24 (53) 10 (22)
Primary surgeon *n* (%)	
Resident Senior registrar Chief physician	5 (11) 8 (18) 32 (71)
No. of people present, median (min.–max.)	
Sign-in Time-out Sign-out	4 (2–7) 6 (3–8) 5 (4–7)
No. of people focused, median (min.–max)	
Sign-in Time-out Sign-out	3 (1–6) 5 (2–7) 3 (2–6)
Initiation of checklist completion based on HCP role, *n* (%)	
Sign-in (*n* = 45)	
Anesthesiologist Gynecologist Nurse anesthetist OR nurse	5 (11) 2 (4) 15 (33) 23 (51)
Time-out (*n* = 45)	
Anesthesiologist Gynecologist Nurse anesthetist OR nurse	0 (0) 21 (47) 2 (4) 22 (49)
Sign-out (*n* = 36)*	
Anesthesiologist Gynecologist Nurse anesthetist OR nurse	0 (0) 26 (72) 4 (11) 6 (17)
Verbalization of checklist completion based on HCP role, *n* (%)	
Sign-in (*n* = 45)	
Anesthesiologist Gynecologist Nurse anesthetist OR nurse	9 (20) 3 (7) 15 (33) 18 (40)
Time-out (*n* = 45)	
Anesthesiologist Gynecologist Nurse anesthetist OR nurse	0 (0) 43 (96) 0 (0) 2 (4)
Sign-out (*n* = 36)*	
Anesthesiologist Gynecologist Nurse anesthetist OR nurse	0 (0) 34 (94) 0 (0) 2 (6)
Surgical duration (patient arrival to departure, median (IQR)	2 h 41 m (2 h 9 m–4 h 16 m)
Surgical duration (incision to closure), median (IQR)	1 h 30 m (55 m–2 h 49 m)
Sign-in duration, median (IQR), range (min.–max.)	1.8 m (1.1–4.2 m), (0.3–11.8 m)
Time-out duration, median (IQR), range (min.–max.)	0.7 m (0.5–1.0 m), (0.2–3.3 m)
Sign-out duration, median (IQR)	0.7 m (0.4–1.6 m), (0.1–15.6 m)
Students (nursing and medical) present, no. of cases (%)	8 (18)
Relatives present, no. of cases (%)	3 (7)
Re-admission within 30 days, no. of cases (%)	8 (18)

*In nine of the surgeries, no Sign-out was performed

*HCP* healthcare professional, *OR* operating room, *h* hours, *m* minutes, *IQR* interquartile range

Initially, the three observers (KEM, JS, JLS) assessed five cases independently to ensure alignment. When discrepancies or disagreements occurred, the observers discussed them to achieve alignment. KEM and JLS then jointly rated 15 surgeries, and KEM and JS jointly rated 15 other surgeries. Once each surgery was assessed the same alignment approach was applied. Finally, KEM rated the last 10 surgeries. The length of surgery, which was defined as the patient’s arrival in the OR to their departure, ranged from 33 min to 7 h and 49 min.

### General observations

#### Sign-in phase

One key finding from the OR Black Box data was that the Sign-in phase was rarely performed consecutively in a structured way. Several different tasks were done simultaneously; various professional groups preparing own tasks, and with HCPs entering and leaving the room, and the patient arriving in the OR. There were relatives present in eight out of the 45 surgeries. The patient was awake during Sign-in for all of the surgeries.

#### Time-out phase

In the Time-out phase, the team more frequently huddled around the patient, with some of the checklist items (e.g., confirming the patient’s identity, verifying the procedure), being completed more consistently than during the other two pause points.

In general, the composition of the surgical team changed during procedures (e.g., due to breaks, working hours, and/or need for further assistance), and no new Sign-in, Time-out, or Sign-out was performed when a new team member entered the room.

### Compliance scores

#### OR Black Box data

Compliance was 100% for Sign-in and Time-out, while Sign-out was only done 80% of the time.

#### Electronic medical record data

A 100% compliance for Sign-in and Time-out was found. Sign-out was reported in the EMR in 93% of the cases.

#### Quality scores

**Fig. 2:**
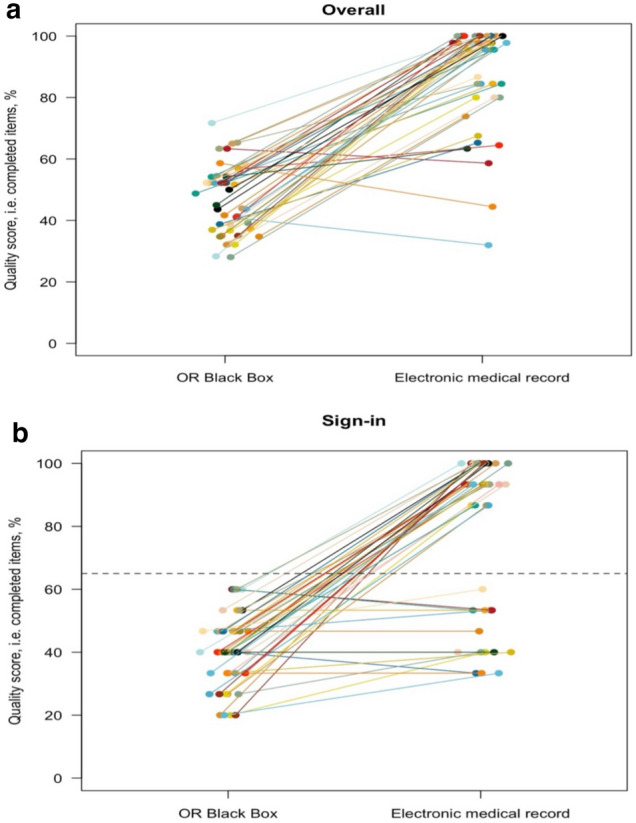
Spaghetti plot of the quality score, i.e., percentage of items completed, for each of the surgeries assessed using video recordings captured by the OR Black Box and data from the electronic medical record. **a** Panel A shows an overall plot of quality scores for all three phases of the Surgical Safety Checklist. **b** Plot for Sign-in quality scores, dotted line illustrates groups of data reported in electronic medical record with quality scores < 65%, and another group of data with scores close to 100%

Figure 2 provides a ‘spaghetti plot’ of the quality score overall (all three phases) and of the Sign-in phase.

Table 2 provides an overview of quality scores for both OR Black Box data and electronic medical record data.

**Table 2:** Discrepancies in quality scores for OR Black Box and electronic medical record data

	Quality score* Items reviewed based on OR Black Box data, % Mean Median (IQR), range (min.–max.)	Quality score* Items reviewed based on electronic medical record data, %, Mean Median (IQR), range (min.–max.)	Mean difference Mean Median (IQR)	Wilcoxon signed-rank test
Overall	47 45 (37–54), 28–72	89 98 (84–100), 32–100	42 46 (34–56)	*p* < 0.0001
Sign-in	40 40 (33–47), 20–60	80 93 (53–100), 33–100	40 47 (13–60)	*p* < 0.0001
Time-out	47 50 (38–50), 25–75	95 100 (100–100), 50–100	48 50 (38–63)	*p* < 0.0001
Sign-out	57 60 (40–80), 20–80	92 100 (100–100), 0–100	33 40 (20–60)	*p* = 0.0001

Scores are reported as a percentage of items completed, overall, and within each Surgical Safety Checklist phase. Differences in mean quality scores between electronic medical record reported and OR Black Box observed data are also shown

*IQR* interquartile range

*The quality score, i.e., the percentage of checklist items reviewed

The quality score, i.e., the percentage of checklist items reviewed and discussed, varied within each SSC phase for both OR Black Box and EMR data (Fig. 2a, Table 2). It was not possible to determine whether items were completed at the correct time using EMR data.

#### OR Black Box data

The overall mean quality score, i.e., the percentage of checklist items reviewed and discussed across all three phases, was 47% (95% CI 43–50). In 8 out of the 45 cases, the patients were readmitted to the hospital within 30 days after the surgical procedure. For these specific cases the mean quality scores for the three phases were as follow; Sign-In 43% (range 20–60%), Time-Out 45% (range 25–62.5%), and Sign-Out 49% (range 20–80%). In one case no sign-out was performed.

#### Electronic medical record data

The overall mean quality score was 89% (95% CI 84–94). Notably, in the Sign-in phase, the quality data was not evenly distributed, instead appearing in two groups, one group with a quality score of 80–100% and the other with a quality score of 0–65% (Fig. 2b). Table 3 divides Sign-in data into a quality score above and under 65%. It gives a detailed overview of individual items completed. While omitted items with a quality score < 65% were primarily related to procedural checks and communication prompts related to the anesthesia team. In terms of EMR data with a quality score > 65%, a completion score per item was mainly > 80%, with the majority of items showing 100% completion.

**Table 3:** Individual item completion data for of the three Surgical Safety Checklist phases in the electronic medical record, divided into quality score < / > 65% for Sign-in

Surgical Safety Checklist phase	Surgical Safety Checklist items according to local practice	Completed items < 65% quality score for sign-in in electronic medical record *n* = 14	Completed items > 65% quality score for sign-in in electronic medical record *n* = 31
		Frequency of completion *n* (%)	Frequency of completion *n* (%)
Sign-in			
Procedural checks	Verification of patient identity	14 (100)	25 (80.6)
	Known allergies	9 (64.3)	31 (100)
	Patient consent	12 (85.7)	30 (96.8)
	Risk of aspiration	0 (0)	31 (100)
	Verification of procedure	14 (100)	31 (100)
	Verification of surgical site	14 (100)	31 (100)
	Risk of airway problems	1 (7.1)	31 (100)
	Relevant, e.g., equipment and implants available in operating room	1 (7.1)	31 (100)
	Indication of antibiotic prophylaxis or not?	0 (0)	31 (100)
	Risk of blood loss (> 500 ml)	0 (0)	31 (100)
	All essential image results displayed in the operating room	0 (0)	31 (100)
	Surgical site marked	11 (78.6)	18 (58.1)
Communication prompts	Anesthesia team present	12 (85.7)	31 (100)
	Complete check of anesthesia machine and medication	0 (0)	31 (100)
	Function check of pulse oximeter on patient	5 (35.7)	31 (100)
Time-out			
Procedural checks	Verification of patient identity	14 (100)	31 (100)
	Verification of procedure	14 (100)	31 (100)
	Verification of surgical site	14 (100)	31 (100)
	Correct patient positioning	12 (85.7)	31 (100)
	Sterile surgical field	9 (64.3)	31 (100)
Communication prompts	Review of anticipated critical events – surgeon	10 (71.4)	31 (100)
	Review of anticipated critical events – anesthesia	9 (64.3)	31 (100)
	Presentation of team	12 (85.7)	31 (100)
Sign-out			
Procedural checks	Specimen labeling	12 (85.7)	29 (93.5)
	Completion of needles, instruments, and sponge count	12 (85.7)	30 (96.8)
	Any equipment problems	11 (78.6)	29 (93.5)
Communication prompts	Review of surgical procedure performed	12 (85.7)	30 (96.8)
	All HCPs – key concerns for recovery and management of patient	11 (78.6)	30 (96.8)

*HCP* healthcare professional

#### Completion of individual checklist items

Table 4 presents completion of individual items each SSC phase for OR Black Box and EMR data. For most items (21 out of 28), a significant difference was found between the two data sources.

**Table 4:** Overview of Surgical Safety Checklist adherence for each phase based on OR Black Box and electronic medical record data

Surgical safety checklist phase	Surgical safety checklist items according to local practice	OR Black Box data	Electronic medical record data	Identical scoring	
		Frequency of completion *n* (%)	Frequency of completion *n* (%)	*p*-value McNemar	Identical responses (95% CI)
Sign-in		45 (100)	45 (100)		
Procedural checks	Verification of patient identity	45 (100)	39 (87)	0.04	87 (73–95)
	Known allergies	37 (82)	40 (89)	0.51	80 (65–90)
	Patient consent	32 (71)	42 (93)	0.009	73 (58–85)
	Risk of aspiration	30 (67)	31 (69)	1	58 (42–72)
	Verification of procedure	27 (60)	45 (100)	< 0.001	60 (44–74)
	Verification of surgical site	21 (47)	45 (100)	< 0.001	47 (32–62)
	Risk of airway problems	15 (33)	32 (71)	0.001	44 (30–60)
	Relevant, e.g., equipment and implants, available in operating room	9 (20)	32 (71)	< 0.001	29 (16–44)
	Indication of antibiotic prophylaxis or not	5 (11)	31 (69)	< 0.0001	33 (20–49)
	Risk of blood loss (> 500 ml)	2 (4)	31 (69)	< 0.0001	36 (22–51)
	All essential image results displayed in the operating room	1 (2)	31 (69)	< 0.0001	31 (18–47)
	Surgical site marked	0 (0)	29 (64)	< 0.0001	36 (22–51)
Communication prompts	Anesthesia team present	45 (100)	43 (96)	0.48	96 (85–99)
	Complete check of anesthesia machine and medication	1 (2)	31 (69)	< 0.001	33 (20–49)
	Function check of pulse oximeter on patient	1 (2)	36 (80)	< 0.0001	22 (11–37)
Time-out		45 (100)	45 (100)		
Procedural checks	Verification of patient identity	45 (100)	45 (100)	-	100 (92–100)
	Verification of procedure	45 (100)	45 (100)	-	100 (92–100)
	Verification of surgical site	34 (76)	45 (100)	0.003	76 (60–87)
	Correct patient positioning	0 (0)	43 (96)	< 0.0001	4 (1–15)
	Sterile surgical field	0 (0)	40 (89)	< 0.0001	11 (4–24)
Communication prompts	Review of anticipated critical events – surgeon	31 (69)	41 (91)	0.016	69 (53–82)
	Review of anticipated critical events – anesthesia	10 (22)	40 (89)	< 0.0001	29 (16–44)
	Presentation of team	3 (7)	43 (96)	< 0.0001	11 (4–24)
Sign-out		36 (80)	42 (93)		
Procedural checks	Specimen labeling	27 (75)	41 (98)	0.23	69 (52–84)
	Completion of needles, instruments, and sponge count	15 (42)	42 (100)	< 0.0001	44 (28–62)
	Any equipment problems	3 (8)	40 (95)	< 0.0001	11 (3–26)
Communication prompts	Review of surgical procedure performed	34 (94)	42 (100)	1	86 (71–95)
	All HCPs – key concerns for recovery and management of patient	23 (64)	41 (98)	0.05	53 (35–70)

*HCP* Healthcare professional

#### OR Black Box data

We found that some SSC items were rarely covered. The most commonly omitted items were in the Sign-in phase: surgical site marked/all essential image results displayed in the OR/complete check of anesthesia machine and medication/function check of pulse oximeter on patient. In the Time-out phase; correct patient positing/sterile surgical field. In the Sign-out phase: any equipment problems.

The OR Black Box data showed that, even though the pulse oximeter and anesthesia machine checks had been completed, they were not verbally confirmed and were thus marked as not completed. We also found that when patient positioning was verbalized, it generally occurred between the patient’s arrival in the OR and before the induction of anesthesia. Even though this item appears in the SSC Time-out phase, it was never verbalized at that point. Although instrument and sponge counts were performed and verbally confirmed in all surgeries, they were completed more than 10 min before final wound closure. Consequently, only 42% of surgeries included the count in the Sign-out phase, raising concerns that checks may occur too early to reliably reflect the status at the time of closure.

#### Electronic medical record data

The item completion rate was 64–100%, for the Sign-in item Surgical site marked, making it the item with the lowest completion rate.

The engagement score was only measured for the OR Black Box data because EMR data did not report people present and focused. The mean engagement score for Sign-in was 86% (range 20–100), for Time-out 77% (range 40–100), and for Sign-out 66% (range 40–100).

The Sign-in process was often unstructured, as checklist items were not verbalized consecutively. The duration of the Sign-in also varied, ranging from 0.5 to 12 min, with people entering and leaving the OR making it challenging to assess the people present and focused. As a result, for all surgeries, engagement scores for Sign-in were assessed when the first item on the checklist, Patient identity, was verbalized. Engagement scores for Time-out and Sign-out, in contrast, covered all checklist items verbalized during the respective phase.

### Associations between outcomes of interest

#### OR Black Box data

We explored potential associations between quality scores and engagement scores, though most findings were not statistically significant.

For example, when adjusted for number of people present in the OR, the overall quality score increased by 0.1 (95% CI − 0.2–0.4, *p* = 0.4) per one-point increase in engagement score. Divided into each SSC phase, the Sign-in quality score decreased by 0.1 (95% CI − 0.22–0.1, *p* = 0.4) per one-point increase in engagement score. The time-out quality score increased 0.2 (95% CI − 0.04–0.38, *p* = 0.12) per one-point increase in engagement score. Last, the Sign-out quality score increased 0.4 (95% CI 0.1–0.8, *p* = 0.02) per one-point increase in engagement score (Table S1).

The overall quality score increased by 0.05 (0.01–0.09, *p* = 0.02) per each additional one minute of surgery from incision to closure. Likewise, the overall quality score increased by 0.04 (0.01–0.08, *p* = 0.02) per each additional one minute from patient arriving to leaving the OR. Examination of the association for each phase did not result in any significant findings.

## Discussion

The study demonstrates clear performance gaps in *how* SSC was executed across the 45 consecutively performed surgeries. We assessed its execution in a gynecological OR at a large tertiary hospital in Denmark based on OR Black Box data and patient EMR data. This is the first study to compare SSC adherence for the same surgical procedure using recorded OR Black Box data and reported EMR data. Execution of SSC was assessed using three metrics: compliance, quality, and engagement. This approach is based on the assumption that it yields a more detailed understanding of *how* SSC was executed, whether it was used as intended, and gives a realistic picture of ‘work-as-done (i.e., how work is actually done)’ as opposed to ‘work-as-imagined (i.e., how it is thought/imagined work is done)’ [34]. This contrasts with the approach solely examining *if* the SSC was executed based on a yes–no response, like most EMR data reports.

This approach, which resulted in multiple variables and highly complex data, provided a valuable opportunity to gain detailed insights into SSC adherence and where it can be improved. Moreover, the data produced is comparable and can be used with other hospitals using the same approach [17, 18, 32].

Overall, the study data indicates that the SSC was used with perfect consistency throughout the Sign-in and Time-out phases, achieving 100% compliance, whereas the Sign-out phase reached 80% compliance. Notably, the surgical team rarely performed the checklist while huddling with a clear team leader, and with all HCPs focused and actively participating. This was reflected in the average engagement (focused HCP) score of 76% (range 45–84%) and in the diversity of who initiates and completes the SSC. As stated in the original WHO checklist [3], all team members should be present in the OR, but based on our OR Black Box data, the gynecologists only participated in the Sign-in phase in two of the cases.

The quality scores, i.e., the percentage of checklist items completed, also indicated that the checklist was not used as intended, as many of the items were not verbalized. We also observed significant discrepancies in quality scores between what was observed based on video data versus what was reported in the EMR for each surgery: 47% (95% CI 43–50) for the video observations vs. 89% (95% CI 84–94) for the EMR reports. Other observational studies have also identified variations in the execution of the checklist [17, 19, 20, 23, 32] and differences in performance between observed and reported use [27, 35]. Closer examination of the completion of individual checklist items showed inconsistent item completion with rates ranging from 0 to 100% across the three phases based on the observational data, while reported EMR data ranged from 64 to 100%. Similar to other studies [18, 21, 35, 36], critical SSC items were not verbalized. More specifically, OR Black Box data showed that key items, such as patient positioning, risk of blood loss, check of anesthesia machine and pulse oximeter, antibiotic prophylaxis, and team presentation, were verbalized during less than 12% of the surgeries.

Although the SSC was introduced over a decade ago and numerous studies have found positive effects on patient outcomes [5–7, 9, 37, 38], the debate, starting after Urbach et al.’s study [11] from Ontario, Canada, remains highly relevant. The survey-based study, which included 101 hospitals and was designed as a three-month pre- and post-implementation SSC evaluation, showed a high self-reported SSC use (98%), but found no impact on the complication or mortality rate following the mandated use of the SSC. In response to this study, several challenged its results and limitations [39–44]. It was particularly questioned that the study was only based on self-reported data (which often shows a much higher level of compliance), most of the hospitals included in the study used an unmodified version of the WHO SSC (lack of involvement of end-users, mandated use from the Ministry of Health and Long-Term Care), no formal training in how the SSC should be used was conducted (only educational material was handed out), and the evaluation period was considered short. In this debate, Leape stated that even though the checklist may seem like a simple and straightforward tool with mainly “yes” and “no” questions, it is far more complex than that, as it depends on the behavior of HCP and the culture inside the OR, and it is not just a matter of ticking-off boxes [42]. Execution of the SSC requires getting the surgical team together at specific time points during surgery, ensuring that all team members are engaged and focused, and that all checklist items are being verbalized and confirmed. If the SSC is performed in a team huddle in a streamlined process and a psychological safe environment, where items are completed consecutively in a structured way, it will most likely reduce the risk of missing items and improve proper communication of individual checklist items to the whole team, e.g., verbalization of counting of sponges and instruments. Pausing briefly and slowing down for a short time before starting the procedure may also establish the time and space needed to properly execute the checklist [45]. Doing this right at all three phases of the checklist requires more than just mandating the use of the checklist; it may require behavioral change among HCPs, which can be a challenging undertaking [46], as it may involve pointing out that the way things are done is not how it should be done. Promoting a change in how the SSC should be executed requires training [32], time [42], understanding of the context within which it should be carried out [47], and buy-in from all HCPs participating in the surgical procedure [15]. It is well stated that HCP groups perceive SSC differently [12, 48] and have received different levels of training in the clinical use of the SSC [48]. One of the most common barriers to implementation is resistance from staff (mostly surgeons) [49], which is why changes must accommodate the perspectives of all important stakeholders to ensure buy-in and its consistent use. However, when such changes are done, it should be done in a psychologically safe environment, where everyone feels safe speaking up about observations and concerns [50, 51].

Several other key factors essential to the success of the checklist have been identified [12, 49, 52, 53]. Creating ownership among frontline users may involve that all HCPs have a clear understanding of, for instance, who takes the lead in executing the SSC, the most appropriate time to complete it, which HCPs are actively participating, when all non-essential activities cease, who reports its execution, and what is reported. Having a shared understanding of *who* should initiate and complete the checklist may decrease the risk of misunderstandings. Since OR nurses are present from the patient’s arrival in the OR until the patient leaves, and are the only staff group authorized to mark the checklist items in the EMR, they are uniquely positioned to support checklist completion. While it may seem relevant that the OR nurses initiate the checklist, doing so effectively requires training and active support from all other HCPs involved. Further involving HCPs in shaping the checklist so that it matches the local context and workflow, and would most likely lead to a stronger buy-in by all stakeholders [53, 54] and create a shared understanding and awareness of the checklist. Prioritizing adequate interprofessional training is essential to succeed in achieving adherence and can potentially lead to improved execution of SSC [12, 32] and better patient outcomes [10]. Well-trained staff provides a solid foundation for good teamwork from the moment the patient arrives in the OR. Research indicates that a lack of teamwork during the initial Sign-in phase increases the likelihood of poor teamwork throughout the surgery [55], which underscores the need to focus on interprofessional training.

### Future perspectives

Based on our results, it is evident that there is a need for a change, such as an educational intervention to streamline the execution of the checklist and ensure a shared understanding of how to use it and its intended use among all relevant stakeholders, e.g., HCPs and management. The failure to verbalize key items of the checklist can have critical implications for the patient, when rapid action is required or to prevent any complications. Failing to administer, e.g., antibiotics at the right time, may increase the risk of surgical site infection [56]. As observed through OR Black Box data, the checklist item “administration of antibiotics” was verbalized in only 11% of cases. It is therefore relevant for future studies to investigate whether antibiotics were actually administered and whether the patient developed an infection after the surgical procedure.

The majority of cases in this study were elective procedures performed during the daytime, as the OR Black Box at our institution only captured data from 7 AM to 5 PM while conducting this study. The OR Black Box now captures data for 24 h in our department, which allows for future studies to include acute procedures.

This study can be used to focus on and improve the evaluation and awareness of *how* to enhance SSC use and report reliable data for HCPs who work in the OR and with SSC. One possible future direction is to identify what interventions and types of training are needed to ensure that SSC is used correctly, but also how often refresher training is required and obtaining a greater understanding of what happens inside the OR and what behavioral change is needed, which requires additional qualitative work.

### Strengths

This study includes reported EMR data and observed OR Black Box video data on how SSC is executed. One of the strengths of the OR Black Box system is its ability to provide a transparent, realistic, and unbiased assessment of what happens in the OR without being present in the OR. This feature provides valuable insights into the complex factors affecting the use and reporting of SSC, but also knowledge of where performance gaps exist, in addition to creating a foundation for communicating openly about where to improve, identifying areas for targeted interventions, and ensuring adequate SSC training.

### Limitations

One of the limitations of this study is that it was conducted in a single gynecological OR and a limited number of surgeries (*n* = 45) were assessed, which limits its generalizability. The Hawthorne effect, i.e., improvement in team members’ performance due to being observed, is a risk in all observational studies [57]; however, the OR Black Box system had been in use for nearly 12 months at our department [30] when this study took place, likely lessening the risk of the Hawthorne effect occurring. Another limitation is that Sign-in items were sometimes performed before the patient arrived in the OR, creating the risk of a false sense of what had been completed.

## Conclusion

Although SSC appears to be a simple and straightforward tool, using it is a complex task, and it is not always used as intended. Our study found a clear discrepancy in how SSC was executed by comparing EMR data and OR Black Box data, emphasizing the need for interventions to improve how the SSC is used and to mitigate the gap between ‘work-as-done’ and ‘work-as-imagined’. The EMR data showed a much higher percentage of items completed, but this was not entirely confirmed by the OR Black Box data, making it a valuable tool for assessing *how* SSC was performed. Thus, our results highlight the importance of not relying solely on EMR data, or other observations prone to biases to monitor SSC adherence.

## Supplementary Information

Below is the link to the electronic supplementary material.

Supplementary file1 (DOCX 486 KB)
