# Supplementary material for: Does the Surgical Safety Checklist need a co-pilot? Comparing adherence in gynecological surgery through electronic medical records and OR Black Box video observations
Source: Surg Endosc. 2025 Jul 17;39(9):5742–54. doi: 10.1007/s00464-025-11966-0 (PMC12408729; doi:10.1007/s00464-025-11966-0)
Supplement: Supplementary file 1 — Supplementary file1 (DOCX 486 KB) [file 464_2025_11966_MOESM1_ESM.docx]

**SUPPLEMENTARY**

The study “*Does the Surgical Safety Checklist Need a Co-Pilot? Comparing Adherence in Gynecological Surgery Through Electronic Medical Records and OR Black Box Video Observation*“ assessed adherence to the WHO SSC during gynecological surgeries using the modified checklist version to suit local conditions and workflows in our department. Video observed data and data reported in patient’s electronic medical record were compared.

**FIGURE S1:** WHO Surgical Safety Checklist and modified local version.


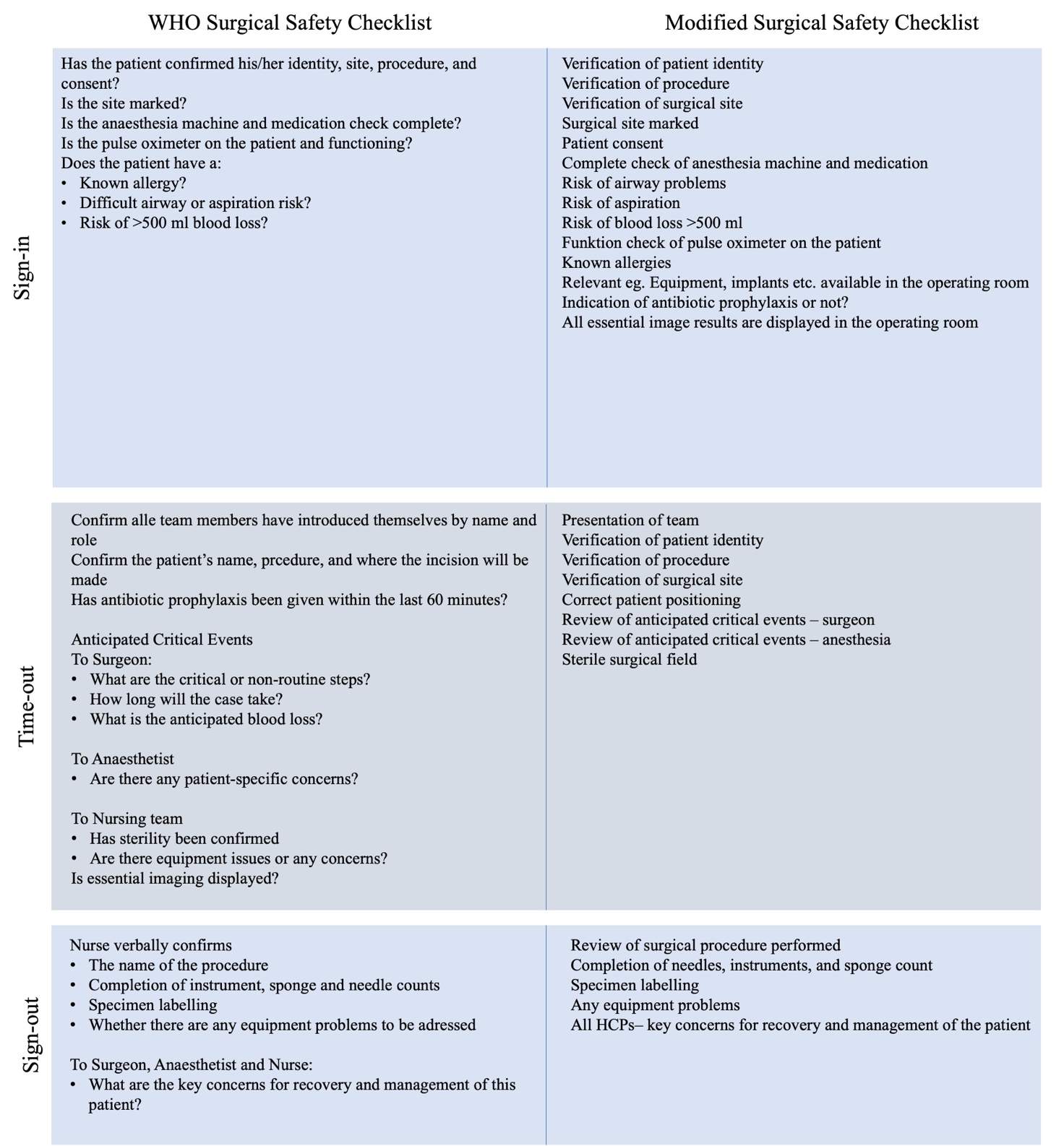


TABLE S1 – Presentation of the association between quality (percentage of items completed) and engagement scores (percentage of people focused out of people present in the operating room) based on observational OR Black Box data

|  | **Quality score**  **Items reviewed based on OR Black Box data, %,**  Mean,  Median (IQR) | **Engagement score**  **People focused/people present based on observed OR Black Box data, %,**  Mean (SD), Median (IQR)  Range | **Association between quality and engagement,**  (95% CI) p-value | **Association between quality and engagement**  (95% CI)* p-value |
| --- | --- | --- | --- | --- |
| Overall | 47  45 (37–54) | 76  76 (68–86)  45–94 | 0.2 (-0.1–0.5),  p=0.3 | 0.1 (-0.2–0.4) p=0.4 |
| Sign-In | 40  40 (33–47) | 86  100 (71–100)  20-100 | -0.1 (-0.2–0.1) p=0.2 | -0.1 (-0.2–0.1) p=0.4 |
| Time-Out | 47  50 (38–50) | 77  80 (63–86)  40–100 | 0.2 (-0.01–0.4), p=0.06 | 1.7 (-0.04–0.4) p=0.1 |
| Sign-Out | 57  60 (40–0) | 66  60 (50–80)  40–100 | 0.4 (0.1–0.8), p=0.02 | 0.4 (0.1–0.8) p=0.02 |

* Adjusted for people present
IQR, Interquartile range; SD, standard deviation.

TABLE S2 – Quality score (percentage of items completed) based on OR Black Box data listed based on the profession that initiated and completed the Surgical Safety Checklist

|  | **Anesthesiologist** | | **Gynecologist** | | **Nurse anesthetist** | | **OR Nurse** | |
| --- | --- | --- | --- | --- | --- | --- | --- | --- |
|  | Initiated | Completed | Initiated | Completed | Initiated | Completed | Initiated | Completed |
| Sign-in  Quality score: mean  Median (IQR) | n=5  43  40 (40–47) | n=9  37  40 (33–40) | n=2  40  40 (37–43) | n=3  42  46 (40–47) | n=15  38  40 (27–47) | n=15  40  40 (27–47) | n=23  41  40 (33–47) | n=18  42  40 (35–47) |
| Time-out  Quality score: mean  Median (IQR) | - | - | n=21  49  50 (50–50) | n=43  47  50 (38–50) | n=2  44  44 (41–47) | - | n=22  45  38 (38–50) | n=2  38  38 (38–38) |
| Sign-Out  Quality score: mean  Median (IQR) | - | - | n=26  58  60 (40–75) | n=34  58  60 (40–80) | n=4  45  40 (20–65) | - | n=6  63  70 (45–80) | n=2  40  40 (40–40) |

*IQR: Interquartile Range

TABLE S3 – Mean differences in overall quality score (percentage of item completion) and completion of individual Surgical Safety Checklist items (Completed versus not completed), and in overall engagement score (percentage of people focused out of the people present in the operating room) and completion of individual Surgical Safety Checklist items (Completed versus not completed.).

| **Surgical Safety Checklist phase** | **Surgical Safety Checklist items** | **OR Black Box data** | **Mean difference between overall quality score and checklist item**  **t-test** | **Mean difference between overall engagement score and checklist item** |
| --- | --- | --- | --- | --- |
|  |  | Frequency of completion n (%) | (95 % CI), p-value | (95 % CI)*, p-value |
| **Sign-In** | | **45 (100)** |  |  |
| *Procedural checks* | Verification of patient identity | 45 (100) | - | - |
|  | Known allergies | 37 (82) | 10 (2–18), p=0.02 | 2 (-4–7), p=0.5 |
|  | Patient consent | 32 (71) | 7 (-0.02–14), p=0.05 | 2 (-4–7), p=0.5 |
|  | Risk of aspiration | 30 (67) | 5 (-2–12), p=0.13 | 2 (-4–7), p=0.5 |
|  | Verification of procedure | 27 (60) | 7 (-0.02–13), p=0.05 | 2 (-4–7), p=0.6 |
|  | Verification of surgical site | 21 (47) | 7.1 (1–13), p=0.03 | 1 (-4–7), p=0.7 |
|  | Risk of airway problems | 15 (33) | 5 (-1–12), p=0.12 | 1 (-4–6), p=0.7 |
|  | Relevant, e.g., equipment and implants, available in operating room | 9 (20) | -0.09 (-8–8), p=0.98 | 2 (-3–6), p=0.5 |
|  | Indication of antibiotic prophylaxis or not | 5 (11) | 6 (-5–16), p=0.3 | 1 (-4–6), p=0.6 |
|  | Risk of blood loss (>500 ml) | 2 (4) | 23 (8–37), p=0.003 | 2 (-3–8), p=0.4 |
|  | All essential image results displayed in the operating room | 1 (2) | 5 (-17–28), p=0.65 | 2 (-4–7), p=0.5 |
|  | Surgical site marked | 0 (0) | - | - |
| *Communication prompts* | Anesthesia team present | 45 (100) | - | - |
|  | Complete check of anesthesia machine and medication | 1 (2) | -12 (-34–10), p=0.29 | 2 (-3–7), p=0.5 |
|  | Function check of pulse oximeter on patient | 1 (2) | -5 (-28–17), p=0.65 | 2 (-4–7), p=0.5 |
| **Time-out** | | **45 (100)** |  |  |
| *Procedural checks* | Verification of patient identity | 45 (100) | - | - |
|  | Verification of procedure | 45 (100) | - | - |
|  | Verification of surgical site | 34 (76) | 15 (8–22), p=0.0001 | 1 (-4–7), p=0.6 |
|  | Correct patient positioning | 0 (0) | - | - |
|  | Sterile surgical field | 0 (0) | - | - |
| *Communication prompts* | Review of anticipated critical events - surgeon | 31 (69) | 15 (10–21), p<0.0001 | 1 (-4–6), p=0.6 |
|  | Review of anticipated critical events - anesthesia | 10 (22) | 12 (5–19), p=0.001 | 2 (-3–8), p=0.4 |
|  | Presentation of team | 3 (7) | 13 (1–26), p=0.04 | 2 (-4–7), p=0.5 |
| **Sign-out** | | **36 (80)** |  |  |
| *Procedural checks* | Specimen labelling | 27 (75) | 14 (9–19), p<0.0001 | 1 (-5–7), p=0.7 |
|  | Completion of needles, instruments, and sponge count | 15 (42) | 12 (6–18), p=0.0002 | 2 (-3–7), p=0.4 |
|  | Any equipment problems | 3 (8) | 13 (0.2–26), p=0.05 | 2 (-3–7), p=0.4 |
| *Communication prompts* | Review of surgical procedure performed | 34 (94) | 9 (2–16), p=0.01 | 2 (-4–7), p=0.5 |
|  | All HCPs – key concerns for recovery and management of patient | 23 (64) | 14 (8–19), p<0.0001 | -0.003 (-5.3–5.2), p=1.0 |

* Adjusted for people present.
